# An Improved Fabrication Technique for the 3-D Frequency Selective Surface based on Water Transfer Printing Technology

**DOI:** 10.1038/s41598-020-58657-5

**Published:** 2020-02-03

**Authors:** Maxime Harnois, Mohamed Himdi, Wai Yan Yong, Sharul Kamal Abdul Rahim, Karim Tekkouk, Nicolas Cheval

**Affiliations:** 1 Institut d’électronique et des Télécommunication de Rennes, UMR CNRS 6164, Université de Rennes 1, Campus de Beaulieu, 35042 Rennes Cedex, France; 2 Department of Electrical Engineering, University of Twente, 7500AE Enschede, Netherlands; 3 Wireless Communication Center, Universiti Teknologi Malaysia, 81310 Johor Bharu, Malaysia; 4 Thales DMS Centre Charles Nungesser 2 Avenue Jean D’Alembert, 78995 Elancourt, France

**Keywords:** Electrical and electronic engineering, Sensors and biosensors, Design, synthesis and processing, Surface patterning

## Abstract

Manufacturing an array of high-quality metallic pattern layers on a dielectric substrate remains a major challenge in the development of flexible and 3-D frequency selective surfaces (FSS). This paper proposes an improved fabrication solution for the 3-D FSS based on water transfer printing (WTP) technology. The main advantages of the proposed solution are its ability to transform complicated 2-D planar FSS patterns into 3-D structures while improving both manufacturing quality and production costs. WTP technology makes use of water surface tension to keep the thin metallic patterns of the proposed FSS floating flat with the absence of a solid planar substrate. This feature enables these metallic FSS patterns to be transferred onto 3-D structures through a dipping process. To test the effectiveness of the proposed technique, the FSS was designed using computer simulation software Microwave Studio to obtain the numerical performance of the FSS structure. The WTP technology was then used to fabricate the proposed FSS prototype before its performance was tested experimentally. The measurement results agreed well with the numerical results, indicating the proposed manufacturing solution would support the development of complicated 3-D electronics devices, such as conformal antenna arrays and metamaterials.

## Introduction

A frequency selective surface (FSS) is a periodic array structure designed using either radiating or non-radiating elements^1^. The FSS can be function as either a band-pass or band-stop filter. FSSs are widely used as radomes which function as spatial filters shielding unwanted electromagnetic signals^2^. FSSs are also commonly used in radar systems for radar cross-section reduction^3^. To ease the integration of FSS with other devices and structures while extending FSS applicability, FSS with flexible characteristics are required^4^. To date, several studies have investigated the manufacturing techniques for flexible electronic components^5–8^. Despite this, most of these technologies remain focused on the fabrication of small-scale electronic devices. To improve the marketability of these electronic components, more research works is needed to create a low-cost, high-quality technique for fabricating flexible, large-scale and 3-D electronics devices.

To this end, numerous investigations in the field of conformable electronics have focused on the fabrication solutions in realizing the flexible and stretchable electronic components^9–14^. Previous research has established that these flexible and stretchable electronics can be realized by replacing the planar rigid substrate with the conformable substrate such as Polyethylene Naphthalate (PEN), Polyimide (PI), or a stretchable rubber known as Polydimethylsiloxane (PDMS)^9,10^. In the design of the flexible electronic components, previous research works have proven that substrate thickness plays a crucial role in determining the conformability behaviour of flexible electronics^12^. Indeed, when a thinner substrate is used, electronic devices can conform to another structure more easily^13,15^. Salvatore *et al*.^15^ demonstrated the possibility of developing a flexible electronic device with extraordinary bending performance using a micrometre-thick polymeric substrate, but their proposed solution remains controversial given industry demands for large-scale manufacturing. Although the proposed solution in^15^ would resolved the integration issue of highly flexible electronic devices, the manipulation of an ultra-thin substrate remains challenging, especially in the field of large-area electronics.

Another interesting solution allowing the integration of electronic components with 3-D structures is developing electronics components with the stretchable features. Unlike flexible electronics components, stretchable electronics may be bent or expanded in-plane. Such electronics can be fabricated using stretchable interconnects (e.g. buckled or serpentine design)^16^, liquid metal^17^ and intrinsically stretchable materials^16,18,19^. Several electronics, such as implantable neuro monitors and stimulators^20^, optoelectronic devices^21^ and conformal photovoltaic devices^22^, have been fabricated utilizing these technologies, but the fabrication of large-scale electronic devices, such as FSSs, remains impractical due to the time consuming in the fabrication which also results in high fabrication cost. To address the large-scale manufacturing issue for 3-D devices, alternative solutions, such as aerosol jetting^23^, direct printing^24^ and 3-D moulded interconnected devices^25,26^, have been proposed. While these proposals offer solid support for large-scale manufacturing of the metallic and insulator layers of electronic devices^26^, they remain immature and unable to fabricate complex electronics and electronic components that would serve actual industrial applications^26^.

Recently, a novel water transfer printing (WTP) technology was introduced for the fabrication of small-scale electronic devices^14,27–30^. WTP, also known as hydrographic printing, is also commonly used in graphic art^31^. WTP allows the transfer of 2-D planar designs onto 3-D surfaces of any shape. In addition, the fabrication of flexible electronic devices using WTP can be realized easily using water-soluble substrate and water^14^, removing the need for expensive facilities, such as custom made 3-D moulds and thermoforming machine.

In this paper, we propose to evaluate WTP compatibility by fabricating the array structure of FSS devices using a proposed WTP technology. To demonstrate the advantages of the proposed technology, we shall provide a comparative study between WTP and established thermoforming fabrication technology. The design of a FSS element, fabrication set-up, fabrication process and FSS characterization results will be presented. The proposed WTP is expected to be an attractive solution for the manufacturing of large-scale 3-D electronic devices and a potential manufacturing candidate for the fabrication of the 5G massive MIMO (Multiple-Input Multiple-Output) antenna arrays.

## Results

### FSS design

FSSs are periodically-arranged conductive patterns fabricated on an insulator surface to provide band-pass or band-stop characteristics^4^. An FSS is commonly used as a radome or for radar cross-section reduction^1^. FSSs are usually installed on structures with varying designs, such as buildings or aircraft bodies. FSS performance is highly dependent on how its metal is deposited on the insulator surface^2^, making the dimensions of that metallic layer a key performance parameter. Therefore, a low-cost manufacturing technique with consistent and high-resolution performance is desirable for the FSS fabrication.

**Figure 1:**
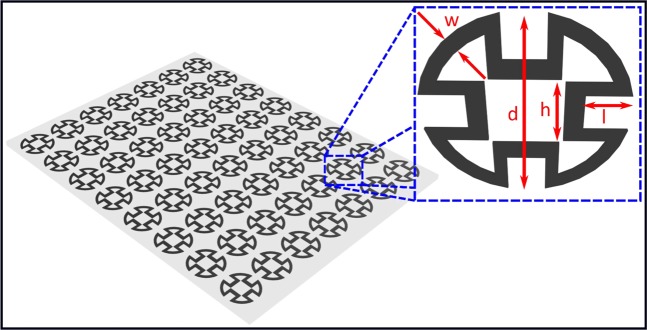
The proposed FSS unit cell design of the CRL; *d* = 7.2 mm, *l* = 1.2 mm, *h* = 1.6 mm and *w* = 0.5 mm.

In this work, a single-band convoluted ring loop (CRL) FSS, illustrated in Fig. 1, was designed to provide filtering characteristics at X-band performance. The assigned FSS unit cell size was 7.2 × 7.2 mm.

The proposed CRL pattern is convoluted from the ring loop element by introducing a square slot at each of the 90° point of the conventional ring loop element^4^. By doing so, the overall dimension of the unit cell was reduced by 33%. With more compact dimensions, more FSS unit cells can be accommodated over the limited space structure to produce a stable frequency response performance^2^.

### Fabrication of the 3-D FSS

We demonstrated two types of fabrication techniques on the manufacturing of the 3-D FSS prototype: thermoforming and WTP technology.

#### Thermoforming fabrication technology

Multiple studies have confirmed that thermoforming technology performs well during the fabrication of large-area interconnected circuitry^26^. The thermoforming process develops the 3-D shape of proposed components, using patterns fabricated on a planar substrate. The thermoforming process is also applied on the planar circuit afterward to give it the desired 3-D shape.

**Figure 2:**
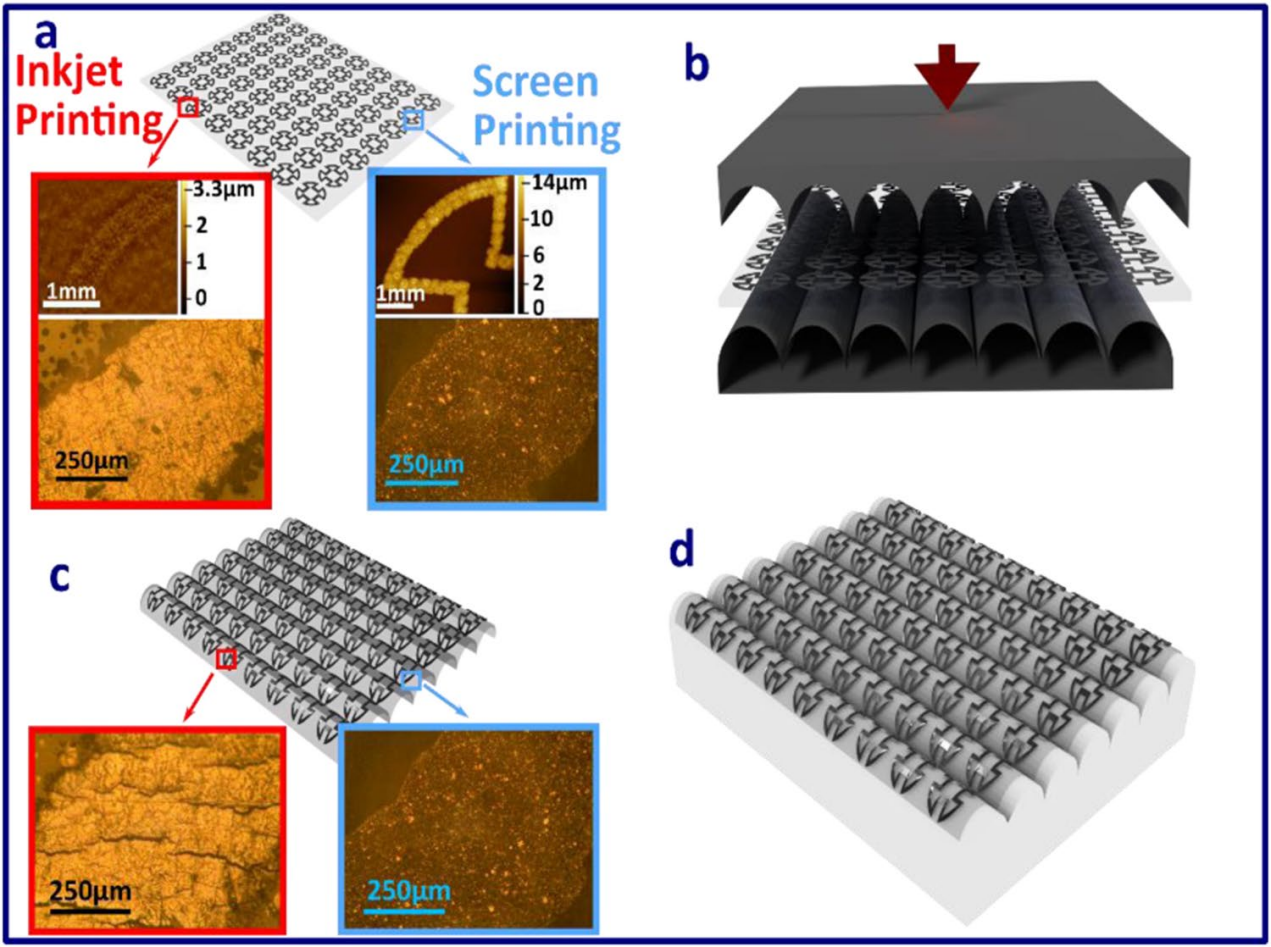
3-D illustration of the fabrication of a 3-D FSS, based on thermoforming process. (**a**) Conductive metallic FSS patterns arranged in an array are patterned onto a polyetheylene terephthalate (PET) substrate based on additive manufacturing techniques (screen printing and inkjet printing); (**b**) sandwiching the 2-D planar FSS between two parts of the mould; (**c**) thermoformed FSS and the optical image of the conductive layer of the FSS after being thermoformed via inkjet and screen printing fabrication techniques; and (**d**) thermoformed FSS bonded onto a 3-D structure.

 Figure 2 illustrates the fabrication process of a 3-D FSS, based on the thermoforming technique. As indicated in Fig. 2(a), two additive manufacturing techniques (drop-on-demand inkjet printing and screen printing) are first employed to fabricate the FSS pattern onto a 2-D planar polyethylene terephthalate (PET) substrate. The PET substrate employed in our experiment was around 0.127 mm thick which can be used easily for bending. The metallic thickness of the FSS pattern was around 12 *μ*m and 3 *μ*m for screen and inkjet printing, respectively. The 3-D profilometry scan and optical images of the conductive layer prior to the fabrication of the thermoforming process are illustrated in Fig. 2(a,c) with the red inserts representing inkjet printing and the blue inserts identifying screen printing. As can be observed from these figures, both of these techniques provided reasonable fabrication quality for the metallic layer.

**Figure 3:**
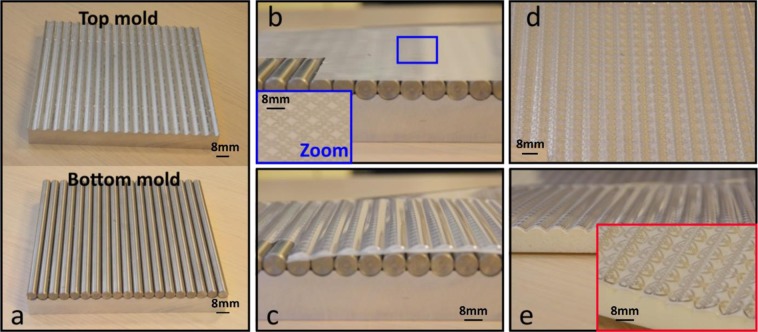
Optical image showing the main thermoforming process. (**a**) Top and bottom part of an aluminium mould; (**b**) the 2-D planar substrate placed on the bottom of the mould; (**c**) the substrates after the thermoforming step; (**d**) the wavy FSS patterns and (**e**) the thermoformed substrate placed on top of the 3-D object. The blue and red insets show higher resolutions of the patterns for screen printing and drop-on-demand printing, respectively.

These planar FSS structures were then sandwiched in between two parts of the 3-D aluminium mould, as shown in Fig. 2(b), and bonded to the 3-D object, as illustrated in Fig. 2(d). Figure 3(a,b) show how an aluminum fold with a waveform shape was employed. The diameter of the waves was fixed at 8mm. To ensure the FSS was patterned onto the expected wave-shaped 3-D structure, the FSS patterns were aligned with the mould. The centre of each FSS unit cell sub-patterns corresponded to the highest location along the z-axis of the wave. By utilizing both mechanical pressing and thermoforming processes, these planar FSS were formed into the 3-D shape shown in Fig. 3(c). Figure 3(e) shows the thermoformed FSS bonded to the 3-D wave-shaped object while Fig. 2(c) exemplifies the 3-D FSS prototype after the thermoforming process based on the aluminium mould. The optical images of the metallic surface condition for these prototypes are shown in Fig. 2(c) as well with the metallic layers of the FSS fabricated using the inkjet printing technique experiencing some cracking after the thermoforming process. This phenomenon has dramatic effects on FSS shielding performance. As such, only the prototype fabricated using the screen printing technique was processed further by attaching it to an object made of epoxy foam coated with a gel-coated layer (see Methods: PVA processing for further details). Fig. 2(e) depicts the final prototype results.

#### Water transfer printing technology

**Figure 4:**
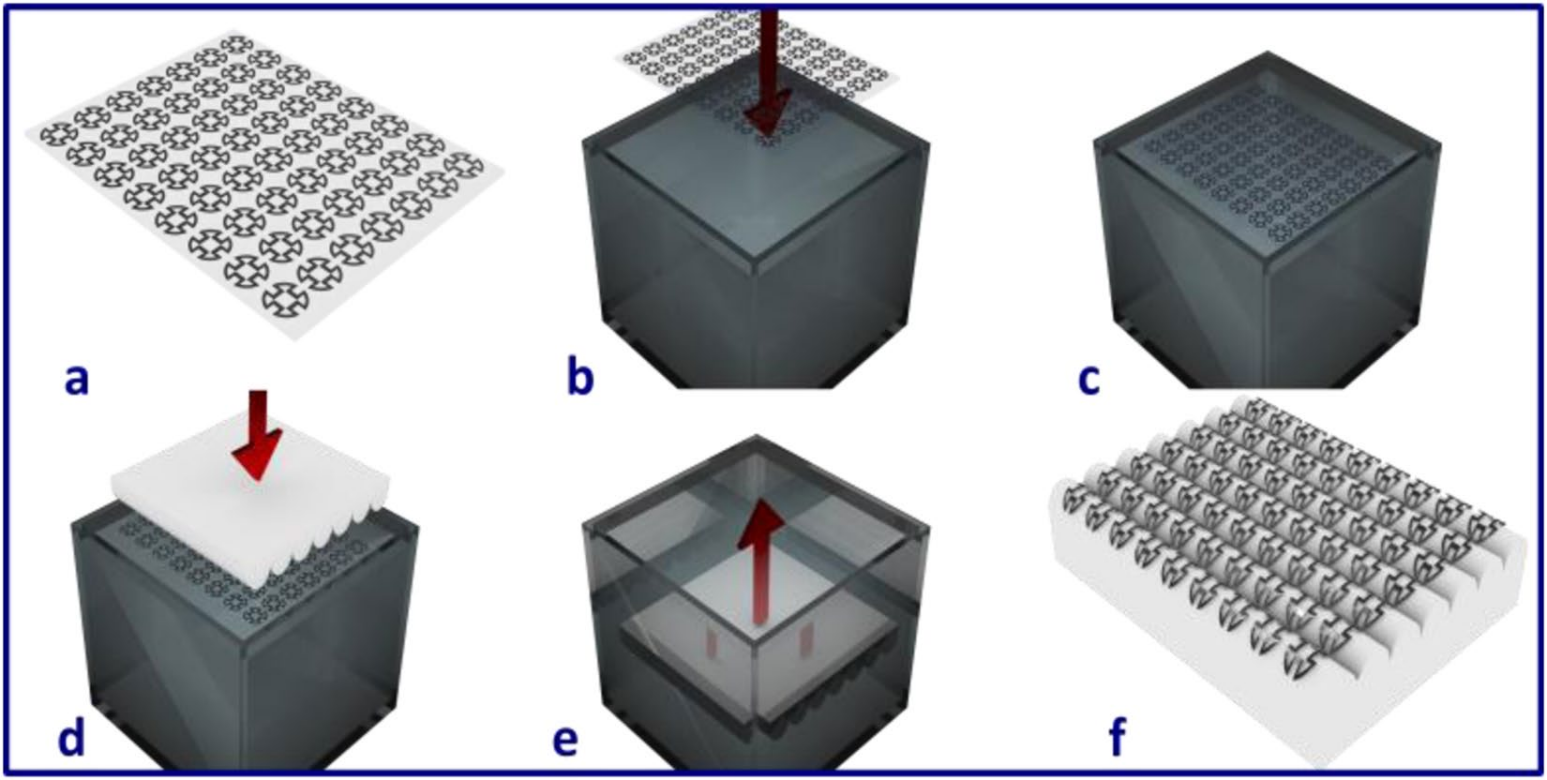
Fabrication of the FSS using WTP technology: (**a**) FSS patterns fabricated onto the 2-D planar PVA substrate; (**b**) depositing the substrate gently onto the water’s surface; (**c**) the substrate floating on the water, allowing the hydrosoluble substrate to dissolve; (**d**) dipping the object through the floating patterns; (**e**) shaking and withdrawing the object; and (**f**) the final object conformally wrapped with the electronics.

**Figure 5:**
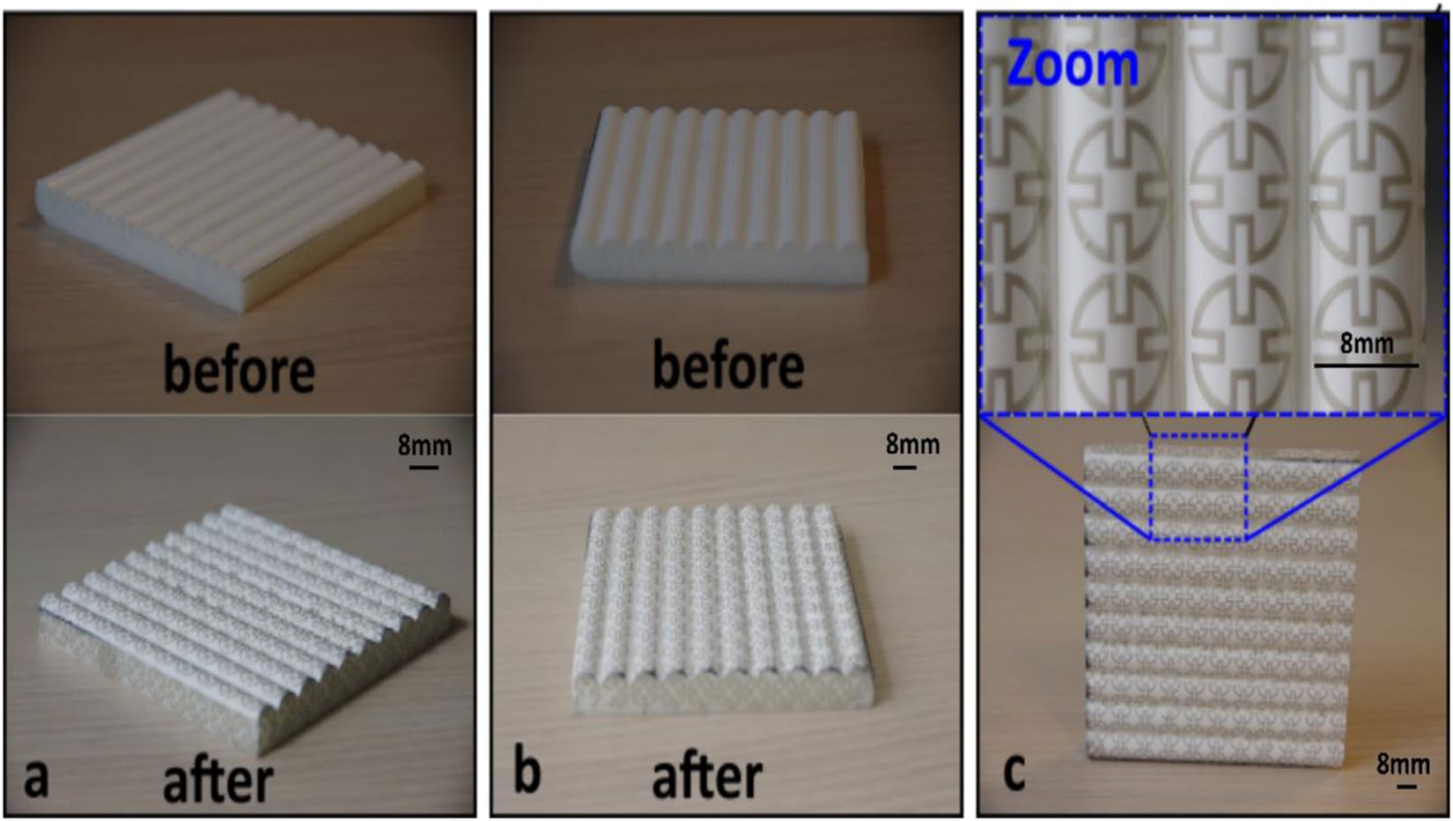
Optical images of the 3-D structure before and after the WTP process for the fabrication of the FSS prototype: (**a**) 45° oriented view, (**b**) 90° oriented view, (**c**) side view showing the top of the wave-shaped structure. The blue inset shows a higher resolution of the sub-patterns of the fabricated 3-D FSS.

The WTP process began with the fabrication of functional films on polyvinyl alcohol (PVA). As only the screen printing technique proved viable during the thermoforming process, it was the only technique used prior to the WTP process. Figure 4 illustrates the 3-D FSS fabrication process utilizing the proposed WTP technology. The PVA substrate with the FSS metallic pattern was first gently deposited on water, as illustrated in Fig. 4(b,c). The PVA substrate was then dissolved in the water, allowing the planar metal patterns to float upon the liquid. The intended 3-D structural recipient of the FSS pattern was subsequently dipped slowly into the liquid through the floating pattern, such that when the object contacted the FSS pattern, as demonstrated in Fig. 4(d), the patterns of the FSS will be transferred onto the intended 3-D shaped. In the dipping process, the liquid applies a resistance to the dip which allowed the pattern to conform to the 3-D structural surface. When the 3-D object was fully immersed, the whole planar pattern was then transferred onto it before it was gently shaken in the liquid and finally extracted as shown in Fig. 4(e). Figures 4(f) and 5 illustrate the final 3-D FSS prototype manufactured utilizing the proposed WTP technology.

The FSS metallic patterns are clearly visible in Fig. 5(c), aligned along the wave shape. To ensure the accuracy of the pattern deposition, a 4-axes motorised positioning equipment, assisted by a camera, was used in the fabrication process (fabrication process was recorded and provided as the Supplemented File).

## Discussion

### Measurement results

**Figure 6:**
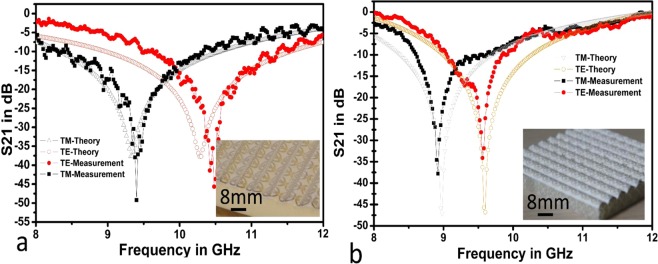
FSS measurements: (**a**) FSS prototype developed onto PET using thermoforming prior to transferring onto a wave-shaped object made of Rohacell; and (**b**) FSS FSS patterns fabricated onto PVA prior to transferring, via WTP, onto a wave-shaped.

To evaluate the performance of the fabricated 3-D FSS prototype, we characterized the prototype experimentally. Figure. 6(a,b) illustrate the comparison of the simulated and measured transmission response for the 3-D conformal FSS prototype fabricated utilizing the thermoformed technique and the WTP technology, respectively, at normal angles of incidence for transverse electric (TE) and transverse magnetic (TM) modes.

As can be observed from Fig. 6(a), the simulation results show that resonance frequency occurs at 10.4 GHz and 9.4 GHz for TE and TM polarizations, respectively. The FSS prototype fabricated via thermoforming produced resonance frequencies of 10.5 GHz and 9.5 GHz for TE and TM polarization, respectively. These frequency shifts are insignificant, proving that this technique provides highly accurate manufacturing dimensions with a prototype more selective than its simulation. This effect could be due to a shape mismatch between the prototype and its simulation or from inaccuracies caused by the meshing stage of the simulation, especially at the junction of two waves.

A similar phenomenon occurred with the prototype fabricated through WTP technology. Although the FSS unit cell dimensions were identical for both prototypes, their simulation results differed, as illustrated in Fig. 6. This phenomenon is mainly attributed to the property differences of the dielectric substrate used in these two technologies, which had a significant impact on the FSS performance. The relationship between the dielectric substrate material and FSS performances has already been discussed by many published works and will be not detailed in this work. In Figure 6(b), the measurement and the simulation results show that the WTP-crafted prototype shielding at 9 GHz and 9.7 GHz, for TE and TM polarization, respectively, with a narrow transmission bandwidth, demonstrating results comparable to the numeric model. Thus, the proposed WTP technology is an alternative manufacturing solution for large-scale 3-D FSS devices.

### Comparing thermoforming and WTP

The main difference between the fabricating technologies relies is the thermoforming process requiring the use of a planar deformable plastic substrate while the WTP process permitted the metallic layer to be deposited directly onto a 3-D structure without the need for a substrate. In terms of conformability, both technologies managed to provide a comparable bending ability when the curvatures of the 3-D structure were along a single axis, as illustrated in Figs. 3 and 5. Due to the dielectric characteristic playing a significant role in determining FSS performance, the WTP process provided a simpler numeric modelling of the FSS devices. In the FSS modelling, the numeric model only requires take a single layer of the dielectric characteristics of the 3-D structure with FSS elements upon it into consideration. Regarding thermoforming, the dielectric effect from both the 2-D planar substrate and the 3-D structure require consideration during the numeric modelling of the FSS devices. To highlight the advantage of the WTP fabrication over the thermoforming process, we fabricated some additional 3-D FSS prototypes on more complex 3-D structures.

**Figure 7:**
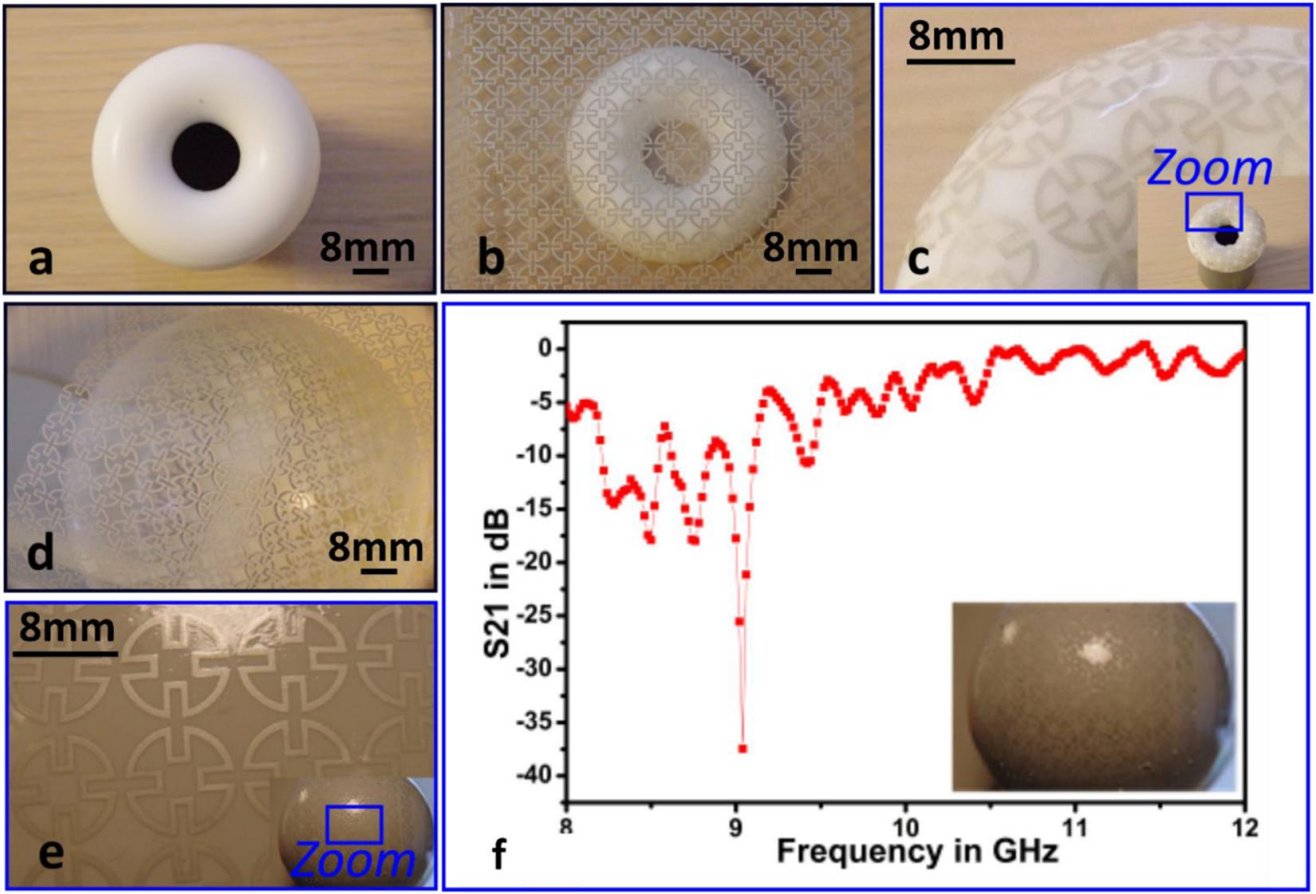
Optical images of the fabricated 3-D FSS on the complex structures: (**a**) a donut-shaped object without FSS deposition; (**b**) a donut-shaped object covered with FSS fabricated onto a PET substrate; (**c**) a donut-shaped object with FSS elements deposited through the proposed WTP technology; (**d**) a semi-spherical object shaped with a PET-based FSS placed on top of it; (**e**) a semi-spherical object shaped with FSS elements deposited through WTP technology; and (**f**) a demonstrated FSS prototype fabricated based on WTP technology onto semi-spherical structure made of PEEK.

Two objects (one donut-shaped and one semi-spherical) were used to fabricate the FSS elements as proof-of-concept prototypes, as shown in Figure 7. As can be observed from Fig. 7(b,d), the themoforming technology was unable to transfer the FSS onto either object (wrinkles and folds were inevitable when wrapping flexible sheets onto 3-D surfaces with two nonzero principal curvatures). As such, the PET-based FSS could only cover the tops of these objects. By contrast, the WTP technology transferred the FSS pattern directly onto both structures, as illustrated in Fig. 7(c,e). Despite this pattern transference onto complex 3-D structures, the pattern distortions can be observed. This phenomenon is exacerbated by the surface of the object when dipped perpendicularly into the liquid, which has been reported in other works dealing with graphic arts^31,32^. This effect is due to the Stokes flow that occurs during the dipping. Methods to resolve it have already been proposed and can be borrowed from the field of conformable electronics^32^.

An FSS was fabricated using the proposed WTP technology on the semi-spherical object because this shape is commonly employed in the design of an antenna radome. The FSS fabricated onto the semi-spherical object was not, however, sufficiently optimised through the simulation. The experimental characterization was performed to demonstrate the band-stop filter ability of such an FSS. As denoted in Fig. 7(f), the fabricated FSS using the proposed WTP technology provided shielding from 8.1–9.1 GHz. Since the FSS performance was not optimized numerically, the measured performance of the FSS prototype was unstable over the operating frequency bandwidth. This is because the FSS performance on the semi-spherical object was unlike the conventional planar FSS, where its performance will be affected by the reflective index of the FSS is varied when it is curved. For a planar FSS, electromagnetic waves travelling through or being reflected would form a planar wavefront. For a curved FSS, a spherical wavefront would be formed as a result of its interaction with the impinging electromagnetic wave, resulting in a change of the reflective index of the FSS. Several bending angles will be evaluated in the future to observe how they affect the performance of a curved FSS. Regarding practical design, the FSS performance of the semi-spherical object requires optimisation prior to fabrication. A comparison of a semi-spherical FSS fabricated via thermoforming was not possible during this study due to fabrication solution limitations.

In this paper, we present the WTP technology as an alternative fabrication solution for 3-D conforming FSS. The conductive fabrication patterns on large-area 3-D structures have been compared between thermoforming and WTP processes. The measurement results have shown significant degradation in the transmission bandwidth, which might be linked to improper FSS design. For the first time, an FSS structure has been being manufactured utilizing WTP technology to form a 3-D curved FSS. Experimentation also demonstrated that it is possible to fabricate an FSS over complex 3-D structures using two proof-of-concept structures (a donut-shaped object and a semi-spherical object) as prototypes. The insights gained from this study may be of assistance to the future fabrication of large-array elements, such as FSS and massive MIMO antennas. This work has also revealed several questions that require further investigation, including the evaluation of possible causes for transmission bandwidth decreases and which resolutions can be supported via WTP technology during the manufacture of compact high-speed communication devices.

## Methods

### Characterization of the FSS prototype

Top-view images and videos were obtained using a PENTAX K70D camera equipped with a ZOOM macro 50 mm (Pentax). Optical microscope images were obtained using a Leica microscope equipped with a digital camera. Transmission responses from the FSS prototypes were carried out in an anechoic chamber. The measurement setup was comprised of two horn antennas (transmitting and receiving antennas) connected to a Rode & Schwarz Vector Network Analyzer with coaxial cables. The FSS prototypes were placed equidistant between the antennas, which were 60cm away from one another, to comply with far-field region conditions^4^.

### PVA processing

The photoresist was first deposited on a glass slide as a sacrificed layer to release the PVA substrate at the end of the fabrication process. A PVA solution (PVA; Mw 9,000–10,000, 80% hydrolyzed from Aldrich) was then prepared by mixing DI water and PVA powder (5: 1 w/w water/PVA) and filtering it through a 0.4 *μ*m filter. The PVA was spin-coated on the photoresist to form a 50 *μ*m-thick layer and baked at 100 °C for two hours. The spin-coating was performed at low rotation velocity of 20 rpm and an acceleration 10 rpm∕*s* ^−1^ for uniform thickness. Metallic thin films were subsequently deposited onto the PVA layer. The PVA substrate was finally delaminated from the glass slide by applying acetone to dissolve the photoresist. The described processing is appropriate for small-area electronics. For large-area fabrication that requires inkjet printing, for instance, the commercial product of PVA laminated on PET films (Aicello, SOLUBLON - 30 *μ*m-thick PVA on 75 *μ*m-thick PET) performs better.

### Characterization of PET

- The PET substrate (AgIC, Japan) was characterized using an open-ended coaxial probe with a vector network analyzer up to 13 GHz.
- Thin film patterning: WTP technology requires that the devices must be processed on a water-soluble substrate. Many approaches to solve this issue have already been proposed^14^.
- In this study, the inkjet printing of silver nanoparticles of AgIC *#*1000 (AgIC, Japan) was performed using a Brother MFC-J430 printer.
- Jetting and drying behaviour of the ink was defined to respect criteria described in previous work^33– 35^.
- Experiments dealing with screen printing were performed at SERIBASE Industrie (Chateau-Gontier, France).

### 3-D wave shaped object fabrication

Aluminium moulds were coated with at least 4 layers of commercial wax. Approximately 1mm thick gel-coating (Sicomin, France) was used as top coated layer. Epoxy foam (Sicomin, France) was applied (1cm thick) and baked at 50 °C overnight.

### 3-Dipping and transfer steps

PVA film was placed on the water’s surface and dissolved before objects were dipped through the floating pattern into the water. Objects were subsequently withdrawn and dried. Van Der Waals force allowed the pattern to stick to each 3-D object.

## Supplementary information

Supplementary Information.
